# Coping and Adaptation in Response to Environmental and Climatic Stressors in Caribbean Coastal Communities

**DOI:** 10.1007/s00267-021-01500-y

**Published:** 2021-07-29

**Authors:** Julia Touza, Carmen Lacambra, Alexandra Kiss, Rosa Mato Amboage, Paula Sierra, Martin Solan, Jasmin A. Godbold, Thomas Spencer, Piran C. L. White

**Affiliations:** 1 Department of Environment and Geography, University of York, Wentworth Way, York, YO10 5NG UK; 2 York Environmental Sustainability Institute, University of York, York, YO10 5NG UK; 3 Grupo Laera, Avenida Carrera 9, No. 113-52 Oficina 1901, PBX 4863358 Bogotá, Colombia; 4 Instituto de Investigaciones Marinas y Costeras (INVEMAR), Calle 25, n° 2-25, Playa Salguero, Santa Marta, Colombia; 5 Ocean and Earth Science, National Oceanography Centre Southampton, University of Southampton, Waterfront Campus, European Way, Southampton, SO14 3ZH UK; 6 School of Biological Sciences, University of Southampton, Highfield Campus, Southampton, SO17 1BJ UK; 7 Cambridge Coastal Research Unit, Department of Geography, University of Cambridge, Cambridge, CB2 3EN UK; 8 Interdisciplinary Global Development Centre, University of York, York, YO10 5DD UK

**Keywords:** Vulnerability, Adaptative capacity, Environmental risk, Socio-ecological resilience, Coastal management, Governance

## Abstract

Cumulative and synergistic impacts from environmental pressures, particularly in low-lying tropical coastal regions, present challenges for the governance of ecosystems, which provide natural resource-based livelihoods for communities. Here, we seek to understand the relationship between responses to the impacts of El Niño and La Niña events and the vulnerability of mangrove-dependent communities in the Caribbean region of Colombia. Using two case study sites, we show how communities are impacted by, and undertake reactive short-term responses to, El Niño and La Niña events, and how such responses can affect their adaptive capacity to progressive environmental deterioration. We show that certain coping measures to climate variability currently deliver maladaptive outcomes, resulting in circumstances that could contribute to system ‘lock-in’ and engender undesirable ecological states, exacerbating future livelihood vulnerabilities. We highlight the significant role of social barriers on vulnerabilities within the region, including perceptions of state abandonment, mistrust and conflicts with authorities. Opportunities to reduce vulnerability include enhancing the communities’ capacity to adopt more positive and preventative responses based on demonstrable experiential learning capacity. However, these will require close cooperation between formal and informal organisations at different levels, and the development of shared coherent adaptation strategies to manage the complexity of multiple interacting environmental and climatic pressures.

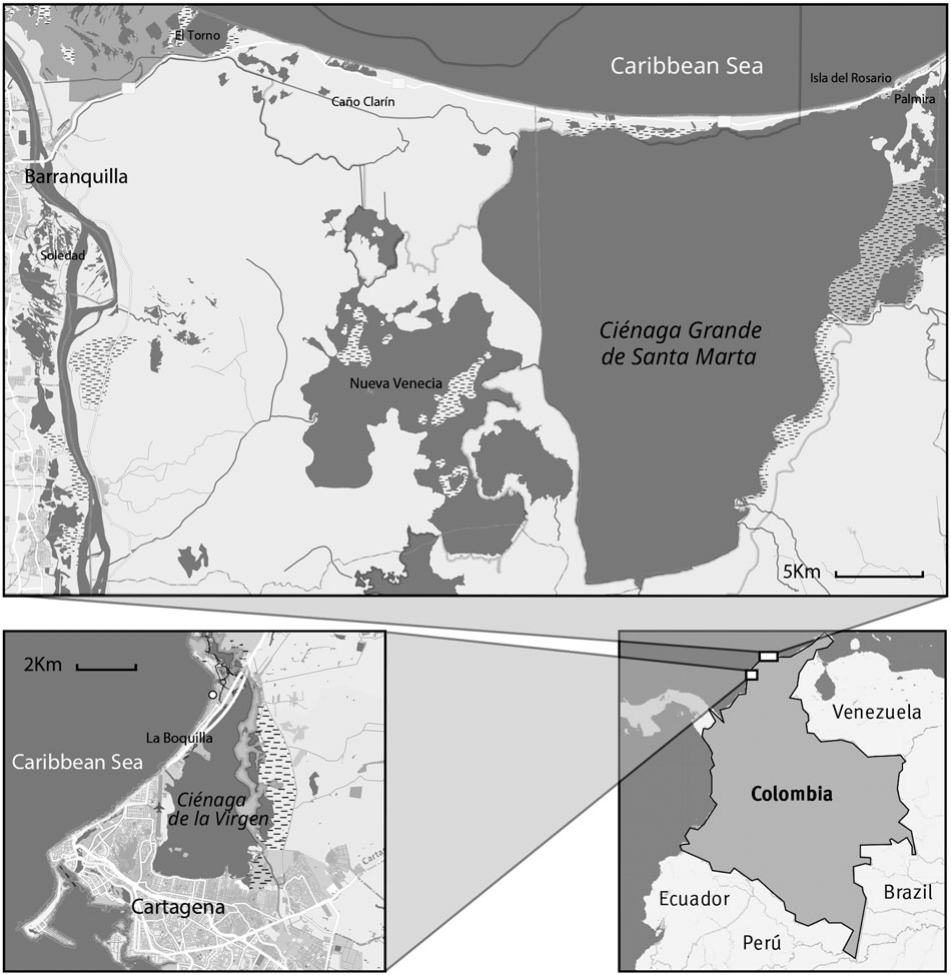

## Introduction

Ecosystems and human communities on low-lying coasts (land below 10 m above mean sea level, contiguous and hydrologically connected to the sea (McGranahan et al. 2007)) are at the forefront of anthropogenic environmental change. They are exposed to a multitude of stressors including climate change, sea-level rise, mass tourism, urban and infrastructure expansion, watershed pollution, sedimentation, habitat fragmentation and overfishing (Allison et al. 2009; Hernández-Delgado et al. 2012). The cumulative effects of combinations of these stressors appear particularly high for the tropical ecosystems of coral reefs, seagrass meadows and mangrove forests (Halpern et al. 2008), but there is considerable uncertainty in projecting the most likely coastal response. Loss of land is already underway worldwide due to anthropogenically induced accelerating coastal erosion (Mentaschi et al. 2018) and will be amplified by increased sea-level extremes and permanent flooding (Hinkel et al. 2013; Oppenheimer et al. 2019). In addition, observed impacts of, and projected increases in, high-intensity extreme events (Knutson et al. 2021) are likely to lead to severe risks to coastal livelihoods and infrastructure by 2050. We are already witnessing extreme increases in temperature and precipitation (Matthews et al. 2017; Wang et al. 2017) that are weakening agriculture potential (González-Orozco et al. 2020). Hence, the combination of high warming scenarios, continued trends in coastal development and low levels of adaptation will lead to challenges for the habitability of many low-lying coastal communities by the latter half of the twenty first century (Magnan et al. 2019) and, even if stabilisation of global temperature is achieved, potential impacts will continue to be experienced for at least several centuries (Nicholls et al. 2018).

Anthropogenic and environmental stressors can have adverse consequences for coastal ecosystem functioning (Hoegh-Guldberg et al. 2018; Bindoff et al. 2019), causing potential irreversible decline in ecosystem services (ESs) (Jackson et al. 2001; MacDougal et al. 2013; Moore et al. 2018). They may also compromise the resilience of socio-ecological systems, with profound impacts on livelihoods, including water supply, river flows, fishery and agricultural yields and human health (Hernández-Delgado 2015; Lauria et al. 2018; Rogers et al. 2019). Moreover, these multiple simultaneous stressors operate over different spatial and temporal scales. Stressors may take the form of extreme weather events with acute spikes in pressure beyond the normal range of variability (e.g. droughts, floods), or as continuously increasing, slow-onset pressures (including chronic pollution or the global environmental change processes of warming oceans, ocean acidification and sea-level rise) or by the interactive combination of both types of effects.

Coping effectively with these multiple stressors has become increasingly important, especially for the poorest and most marginalised people that are more likely to suffer their adverse impacts (Tanner et al. 2015; Whitney et al. 2017). Our aim is to highlight the mechanisms that underpin the vulnerability of low-lying coastal communities when experiencing short term, but high-magnitude, climate variability within longer-term directional environmental change using an evaluation of locally shared knowledge, memories and perceptions. Specifically, we carried out focus groups with small-scale traditional mangrove-dependent communities situated on the margins of two shallow lagoons of the Colombian Caribbean, one of the poorest regions of Colombia (Global Data Lab 2019; DANE-National Administrative Department of Statistics 2012). Case study sites were in: (i) La Boquilla within the Ciénaga de la Virgen, Cartagena, Bolívar Department and (ii) the Ciénaga Grande de Santa Marta (CGSM), near Santa Marta in the Magdalena Department (Map 1). Our analysis of social vulnerability to environmental changes includes the recovery from droughts and floods over annual to decadal timescales, as driven by ocean-atmosphere El Niño–La Niña (ENSO) episodes. In this region, El Niño and La Niña events bring warming and cooling of ocean temperatures, and warming/drought and cooling/flooding, respectively (Poveda and Mesa 1997; McSweeney et al. 2010). In the 2015/2016 ENSO, parts of the Colombian Caribbean coast experienced rainfall at <25% of normal levels between April and October 2015 (El Niño phase) and >150% above normal levels during April to October 2016 (La Niña). These disruptions resulted in substantial economic costs and adverse societal consequences (Comisión Económica para América Latina y el Caribe—CEPAL 2012; Unidad Nacional para la Gestión del Riesgo de Desastres—UNGRD 2016). The La Niña of 2010–2011 affected four million Colombians (9% of the total population), with estimated losses in the country of approximately $US 6–7 billion, equivalent to 2% of national GDP (Comisión Económica para América Latina y el Caribe—CEPAL 2012); some of the worst affected communities were those located along the lower Magdalena River (Hoyos et al. 2013). During El Niño 2015, the Colombian government disbursed more than $US 0.5 billion to confront emergencies (Unidad Nacional para la Gestión del Riesgo de Desastres—UNGRD 2016). As well as ENSO events, the Colombian coast is, very rarely, affected by hurricane landfall. The high winds and rainfall associated with Hurricane Joan (October 1988) resulted in coastal landslides and shoreline erosion, with five deaths, 27,000 people made homeless and $US one billion in damages.

Such extreme episodes, and their impacts, have to be considered within the context of near-future global environmental change. Indications are that some coastal systems show high resilience to droughts, winds and sea-level rise associated with long-term cyclical climatic change, but they respond poorly to short-term extreme events and anthropogenic activity (Urrego et al. 2019). The coastal lagoons between Cartagena and Santa Marta are also under threat from a wide range of slow-onset anthropogenic and environmental stressors, most notably warming (1.7 °C increase by 2070), reduced precipitation (0.8% decline by 2070) and sea-level rise (IDEAM 2015). Under a moderate climate warming scenario (the greenhouse gas trajectory described by Representative Concentration Pathway (RCP) 4.5), regional sea level is expected to rise by 24 cm by 2050 and 52 cm by 2100 (Orejarena-Ronda et al. 2019). There is also a need to consider the possible interactions between events and longer-term change; one such question is how warming trends will impact future ENSO dynamics. While there is no consensus between different climate models on changing ENSO event strength or duration, there is general agreement on the shortening of the period between events (i.e. greater ENSO frequency) (Steinhoff et al. 2015). For the Colombian Caribbean coast, such a change might manifest as an increase in the frequency of extreme rainfall events associated with the La Niña phase of ENSO, with implications for lives and livelihoods in the region.

Social vulnerability can be defined as the contextual social factors that influence the linkage between potential impact, determined by communities’ exposure and sensitivity to natural hazards, and their capacity to adapt to change (Adger 2006; Parry et al. 2007). Specifically, (i) exposure is the magnitude, frequency, duration and/or spatial extent to which a community experiences environmental or social stressors (Burton et al. 1993; Kasperson et al. 2005); (ii) sensitivity is the degree to which the system is affected by the effects of the exposure (Gallopín 2006; Marshall et al. 2010) and (iii) the capacity to adapt (coping capacity, sensu Turner et al. 2003), capacity of response (Gallopín^ 2006^) and adaptive capacity (Smit and Wandel 2006) have been defined as the ability to adjust to stressors, moderate potential damage or cope with the consequences by developing new knowledge and devising effective livelihood practices (Gallopín 2006; Marshall et al. 2010). The adaptation capacity of the beneficiary, however, is not necessarily linearly related to the degree of compromise imposed. Nevertheless, learning from multiple contexts and cases can provide an important foundation for efforts to build appropriate long-term adaptation strategies (Whitfield et al. 2019). Most of the literature measures social vulnerability through quantitative snapshot indicators that highlight the spatial distribution of social inequalities through census data and identify the differential exposure sensitivity and the ability to respond and recover between groups, communities or regions (e.g. Cutter and Finch 2008; de Loyola Hummell et al. 2016; Feindouno et al. 2020). However, this type of analysis is unable to provide the insights into the nature of the interactions between the contextual characteristics (e.g. economy, technology, institutions, governance) and the elements on vulnerability. An increasing number of studies on social vulnerability rely on qualitative approaches using focus groups to capture the richness of the social context (Lizarralde et al. 2020; Aragón-Durán et al. 2020). These vulnerability assessments draw on people’s awareness and experience of the natural hazards they face in the place in which they live. However, these studies tend to include local exposure and adaptation responses to a specific stressor rather than taking an interaction-orientated view among the multiple climatic and biophysical stressors. In fact, the biophysical and environmental aspects of vulnerability are frequently overlooked due to the lack of collaborations across disciplines (Ford et al. 2018). Moreover, vulnerability assessments tend to be for a given sector (e.g. Perez et al. 2020). Therefore, by tending to divide local experiences for particular hazards (e.g. floods) and sectors (e.g. farmers, fishers, urban settlements), and to reflect only a specific moment of time (Ran et al. 2020), the ability of these studies to integrate the interdependencies and feedbacks among environmental, climatic and social drivers of exposure and vulnerability is limited (Simpson et al. 2021). This means that they may fail to inform decision makers on coherent adaptation responses to multiple hazards because the influence of these interactions on behavioural responses, as trajectories of change, is not accounted for when determining overall vulnerability (Li and Ford 2019). In fact, analysis of these interactions has been shown to reveal maladaption trajectories (Eriksen et al. 2021), but also adaptive learning (Naylor et al. 2020). In our approach, members of the communities came together in focus groups to explore their shared vision on the dynamic nature of their dependency on tangible and intangible ESs, associated with the variability in the climate and the ongoing physical degradation of the coastal ecosystem, and their responses to these stressors. The qualitative analysis was based around a conceptual framework that integrate multiple exposures to natural hazards (Perry et al. 2010; Bennett et al. 2016), in order to ground analytically the complexity of the communities’ shared experiences, and the evolving nature of the interactions with the natural environmental over prolonged periods of time. The novel application of an ESs approach (Millennium Ecosystem Assessment—MEA 2005; Diaz et al. 2018) as a broader framing of the discussions in the focus groups was used to aid in this understanding of the mechanisms behind social vulnerability (exposure, sensitivity and responses to impacts), in addition to any specific natural risks. In particular, it encouraged thinking beyond sectoral boundaries and a focus on the entire ecosystem and its multidimensional links in supporting different livelihoods.

## Methods

### Study Sites

The Caribbean coast of Colombia extends for ca. 1800 km from the Gulf of Urabá to the Gulf of Venezuela, the most northerly extension of South America. The marine environment is characterized by surface currents, alongshore sediment transport from the east to the west, a generally low-energy wave climate and a typical tidal range of 20–30 cm (up to 65 cm) (Andrade 2008; Andrade et al. 2013). There is a strong rainfall gradient with the coastline being most arid to the east, where the mean annual rainfall does not exceed 550 mm yr^−1^. Further west, at Cartagena, mean annual rainfall is ca. 900 mm yr^−1^ (HIMAT 1994). The sediment supply to the coast is much less than the littoral transport capacity, with the result that severe erosion characterizes over 50% of the frontage (Rangel-Buitrago et al. 2020). Resulting from these environmental contexts, near sea-level mangrove-fringed lagoons are restricted to broad coastal embayments, fed by inputs of freshwater and sediments. *Rhizophora mangle* (red mangrove) characterizes channel margins and river mouths, fronting mangrove communities dominated by *Avicennia germinans* (black mangrove) with scattered *Laguncularia racemosa* (white mangrove). *Conocarpus erectus* (buttonwood), *Pelliciera rhizophorae* (tea mangrove) and the fern *Acrostichum aureum* are found at the boundaries of the mangrove forest communities, with areas of shrubby *Batis maritima* (saltwort) and *Sesuvium portulacastrum* (shoreline purslane) (Urrego et al. 2013). The average sea-level rise for the Caribbean basin was 2.5 ± 1.3 mm yr^−1^ from 1993 to 2010 (Torres and Tsimplis 2013).

La Boquilla is a coastal district on the northern margin of the city of Cartagena. The Ciénaga de La Virgen is a 30-km^2^ coastal lagoon with a maximum water depth of 2 m and separated from the Caribbean Sea by a 400–800-m wide sand bar (Álvarez-León et al. 2003). Mangrove forest cover is estimated at 775 ha (Villarte Daza et al. 2020). The hydrodynamics of the Ciénaga de La Virgen have been altered in recent decades, first by the construction of the Anillo Vial road around the lagoon between 1984 and 1994, which affected water, sediment and nutrient exchanges (Horta Orozco 2016) and then by excavation of the La Bocana canal through the seaward barrier. This came into operation in the 2000s, restoring water exchange between the lagoon and the sea (Instituto Alexander Von Humboldt—IAVH and Pontificia Universidad Javeriana—PUJ 2015), which, along with a mangrove re-planting programme, increased mangrove coverage on the eastern margins of the lagoon (Villate Daza et al. 2020). Since the 1980s, there has been an accelerated local population growth, due to high birth rates within the community and to the tourism sector expanding towards this area (e.g. the construction of the Las Americas Hotel and the La Boquilla Marina Club in the 1990s (Buitrago Villamizar 2006; Lacambra et al. 2013)). The first reference to artisanal fisheries and small-scale farms existing in La Boquilla dates back to the 1950s. However, declines in natural resources, including fishing catch, pressures caused by informal settlements within the mangroves, and increasing job opportunities in Cartagena are changing the pattern of livelihoods (Buitagro Villamizar 2006). Nevertheless, more than 30% of Cartagena’s population live at, or below, the poverty level, and the lowest-income neighbourhoods, including those located around the Ciénaga de La Virgen, are the most vulnerable to increased flooding (Zamora-Bornachera et al. 2014).

Whilst general wave climates are moderate, the coast near Cartagena is impacted by exceptional wave run-up and sea defence overtopping from long-distance swell waves from the NE, associated with the easterly passage of cold fronts from the Gulf of Mexico across the Caribbean Sea (Otero et al. 2016). Thirty-one such events were recorded in the period 1950–2000 (Nicolae-Lerma et al. 2008, Andrade et al. 2013), on top of a locally very high rate of sea-level rise. Cartagena is thus one of the most flood prone cities in the Caribbean (Reguero et al. 2015), with some areas of the city expected to be 97% flooded by 2100 under a moderate (RCP4.5) climate change scenario (Orejarena-Rondón et al. 2019). The rate of sea-level rise averaged 5.3 ± 0.3 mm yr^−1^ over the second half of the twentieth century, more than twice the Caribbean average, most probably because of a strong contribution from land subsidence (Andrade-Amaya et al. 2017). It seems likely that these land movements are due to sediment compaction in the Castillo Grande coastal spit, which has been subject to extensive urbanization since the 1950s. However, it is not clear if this is a local effect or characterizes the wider Cartagena area. On the coast at La Boquilla, over 7000 homes, and nearly 10,000 inhabitants, are at risk of flooding from the combined effect of sea-level rise and swell wave impacts (City Population 2005; Afanador-Franco et al. 2006). Here coastal risk has been assessed as ‘medium risk’ (Rangel-Buitrago et al. 2020). However, flooding impacts are more severe along the southern and eastern margins of the lagoon where, as at Barrio Policarpa, SW of Cartagena city (Stein and Moser 2014), sea flood events are exacerbated by inadequate urban drainage, lack of storm-water removal and ‘muddy floods’ of polluted sediments (Adams and Castro 2013; Villate Daza et al. 2020).

The CGSM, fed from its western margin by the Magdalena river, is Colombia’s largest coastal lagoon system, covering an area of 3812 km^2^ (INVEMAR 2007) with an average depth of ~2 m (range 0.5 – 9 m, Espinosa-Diaz et al. 2021). It was designated as a Biosphere Reserve in 2000 by UNESCO, has been a designated site under the Ramsar Convention since 1988, and is listed as an Important Bird and Biodiversity Area (IBA). Two national parks have been established within the lagoon system: Salamanca Island Road Park in 1964, and the Santuario de Fauna y Flora in 1977. CGSM is the largest and most productive lagoon in the tropics due to its hydrogeochemical and biological features (Rodríguez-Rodríguez et al. 2018), but is affected by deforestation, land-use changes, water pollution (including eutrophication, hypersalinity and oxygen depletion; Jaramillo et al. 2018; Espinosa-Diaz et al. 2021), freshwater extraction, restriction of water flows and coastal erosion. A total of 25,000 people live across several communities near the lagoon and are heavily dependent on artisanal fisheries for their livelihoods (Blanco et al. 2007). Near the lagoon, there are extensive agricultural activities dominated by banana and oil palm plantations (Aguilera-Díaz 2011). Living conditions are generally poor, with more than half of households (58–73%) having unsatisfied basic needs (DANE—National Administrative Department of Statistics 2012). Between 1956 and 1980, a number of anthropogenic factors affected the region causing severe ecosystem changes. First, the construction of the Troncal del Caribe road, which connects Barranquilla with Ciénaga, interrupted water exchange between the lagoon ecosystems and the sea, producing an increase in salinity and reducing the moisture content of the soil (Aguilera-Díaz 2011; Garay Tinoco et al. 2004). Second, the construction of a road parallel to the Magdalena river (Vía de la Prosperidad, between Palermo and Salamina) and associated dams and embankments reduced freshwater flow into the lagoon system from the Magdalena River (Roederstein et al. 2014). Freshwater channels were re-opened into the wetlands to improve water exchange and flushing capacity in the 1980s (Aguilera-Díaz 2011), although some of the operations intended to increase water connectivity have actually had the opposite effect (Jaramillo et al. 2018). It has been estimated that, by 2005, the cumulative effect of these interventions was that mangrove coverage had been reduced by 253 km^2^ (INVEMAR 2007). Floods and droughts linked to El Niño and La Niña events, and pulses of pollution and sedimentation generated by land-use changes and infrastructure development in the Magdalena catchment (Restrepo et al. 2015), have caused a decline in fishery resources (Ibarra et al. 2014). Government initiatives designed to recover the coastal resources have been sporadic and without previous assessments of their long-term impacts (Rivillas-Ospina et al. 2020).

### Focus Groups

We used eight focus groups (four each in La Boquilla and CGSM) to collect information on the degree of community dependency on the mangrove ecosystem, how periods of heavy rain or drought affect this dependency, and how the community copes with, and responds to, these hydrological and other environmental pressures. Following established techniques (Bunce et al. 2010; Raymond and Robinson 2013; Bennett et al. 2015; McCubbin et al. 2015), we used open-ended questions and activities designed to enable all participants to freely express their experiences, allowing for both focused, detailed information and flexibility in data collection. To avoid communication errors, all conversations took place in Spanish. Members of local organizations, including National Parks personnel at CGSM and NGO staff at La Boquilla, identified members of the community who were interested in contributing to the exercise and had an extensive knowledge of past and present local experiences. The focus groups conducted in La Boquilla included a total of 25 participants, of which most were male, working in eco-tourism or traditional fishing. At CGSM, focus groups included 57 people from six settlements across the lagoon (Caño Clarín, Nueva Venecia, Soledad, Palmira, Isla Rosario and El Torno). Most participants were coastal and/or freshwater fishers (e.g. fishers and shrimp collectors residing in the stilt village of Nueva Venecia, and clam harvesters living in El Torno), although horticulturalists from Caño Clarín were also among the participants. Focus group leaders were given a set of general guidelines and a series of set questions and group activities (Supplementary Material). Participants were asked to describe how the community ‘uses’ the ecosystem, and in general what tangible and intangible benefits they derive from their natural environment. They were also asked to discuss how the community prioritizes and depends on specific benefits; the reasons why some benefits are perceived as being more important than others; and also to consider whether or not the nature of their interaction with the environment has changed across current and previous generations. We also asked participants about their experiences of responses (‘coping methods’) to deal with both extreme weather events (ENSO episodes) and slow-onset pressures, and their views on the effect of the response of the social-ecological system to these coping methods. Participants were asked to consider both positive and negative effects. Each focus group concluded by giving the participants the chance to describe their ‘visions’ or ‘wishes’ or ‘options for management’, reflecting what they expected would happen, or wish would happen, in the coastal lagoon in the near future.

### Analysis of Stressors, Responses and Vulnerability

The outputs from each focus group were analysed using an inductive thematic approach incorporating elements of grounded theory, whereby a framework is developed using research findings rather than by testing a hypothesis (Braun and Clarke 2006; Charmaz 2014). The process for analysing the texts was based on thematic analysis. Three researchers (JT, AK, RMA) independently became familiar with the content via close reading and generated an initial set of themes to code the data. They then searched for theme codes in the transcripts and assigned content to these codes. Following discussion, theme codes were revised and transcripts were re-coded where necessary to establish themes and subthemes, following Braun and Clarke (2006) and Fereday and Muir-Cochrane (2006). Themes were grounded within the literature in order to ‘interpret the information and themes in the context of a theory’ (Boyatzis 1998 p. 11) and were used to inform the vulnerability framework for multiple exposures, as proposed by Bennett et al. (2016). This framework was then iteratively refined to incorporate new and refined codes until no novel thematic content emerged and the framework was stable. Finally, we developed a summary table for each focus group, and each theme, by extracting data from the transcripts under each code and summarizing the themes using appropriate words or phrases verbatim. The integration of the subthemes from the focus groups within the thematic vulnerability framework allowed us to structure the insights gained with respect to perceived exposures, the contextual sensitivity, the impact of environmental and climatic pressures on livelihoods and contrasting coping response strategies.

## Results

**Fig. 1:**
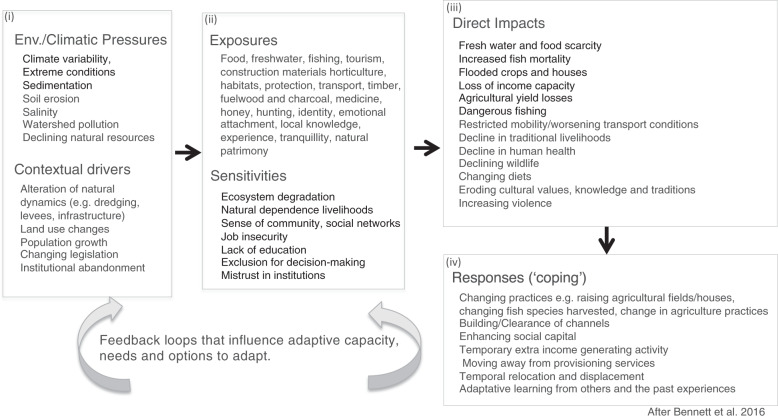
System vulnerability to multiple pressures. Themes and subthemes identified from the focus groups in La Boquilla and Ciénaga Grande de Santa Marta, Colombia

Participants identified a broad range of issues that affect their vulnerability to multiple stressors. Figure 1 presents an overview of themes and subthemes identified from the focus groups. Consistently, with the vulnerability framework of Bennett (2016), the themes are the perceived environmental and climatic pressures and their interconnected contextual drivers (social, demographic, governance factors), and communities’ exposure, sensitivity, impacts and responses. The identified drivers pressures and drivers (subthemes in Fig. 1i), influence communities’ exposure and sensitivities (subthemes in Fig. 1ii), leading to experienced direct impacts (subthemes in Fig. 1iii), and influencing ‘coping’ behavioural responses implemented by communities (for example, raising the level of agricultural fields following flooding; subthemes in Fig. 1iv). The links to long-term adaptation needs and options were also assessed.

### Community Perceptions of Exposure to Environmental and Climatic Pressures

Participants from all communities referred to the multiple pathways of ecosystem function and ES that underpin their livelihoods and way of life, and their exposure to both extreme weather events and ongoing environmental change (Fig. 1i). Communities have a strong sense that contextual human-driven drivers associated with large infrastructure developments, such as new roads and dams, as well as the inadequate draining and maintenance systems of the freshwater channels linking the Magdalena River to the CGSM wetlands, have altered the natural influx of freshwater into the lagoon (Fig. 1i), exposing them to a decline in the key ESs on which their livelihoods depend: ‘before the road [was built] in the year 1955, this region used to be rich in fish and agricultural production, and had, as well, natural richness. Nowadays its production has decreased about 95%’ and ‘the lagoon has now lost its food production capacity’. Thus, the loss of connectivity between the Caribbean Sea and the lagoon, and changing freshwater inputs, were perceived as a slow onset but critical environmental degradation. Participants were aware that the CGSM lagoon is reducing in size and that the waters have stagnated, thus losing the balance from salt and freshwater mixing experienced in the past. This issue was reported to be intensified during summers and drought periods, contributing to increasing salinity and degradation of the lagoon (mangrove forest die-back and fish mortality) and making for a challenging environment in which to maintain livelihoods. These perceptions can be verified by reference to hydrological studies, which have confirmed that the mixing of saline and freshwater in the CGSM depends to a large extent on the influx of freshwater from the Magdalena River. When discharge from the Magdalena is low, there is a greater saltwater intrusion and a high degree of stratification in the water column from a reduction in vertical mixing (Ospino et al. 2018; Restrepo et al. 2018). The long-term reduction in the amount of freshwater entering the system, associated with sedimentation and closure of river channels in the Magdalena watershed (‘the old inhabitants tell that twenty-three rivers of freshwater entered the CGSM, today we have fourteen, the rest was lost’), is likely to have contributed to this problem, with highly stratified conditions becoming more frequent and persistent. Hence, the communities continue to make demands on the authorities to dredge the channels and keep them open, in order to maximize freshwater influx. For example, with reference to one of main freshwater channels that flows into the CGSM lagoon: ‘nowadays Caño Clarín is dry. We have requested the dredging of the stream because a large proportion of the mangroves have died because of the lack of freshwater’ and ‘the lack of freshwater from the streams makes the soil salty and that is affecting us because if the water becomes too salty, animals cannot drink it, people cannot drink it, we cannot irrigate our crops and everything goes bad’. Participants also explained that a good volume and depth of water is necessary for transportation of agricultural products and for personal transport.

Moreover, it was widely recognized by participants from the communities that there is an ongoing critical problem of pollution and contamination in both the Ciénaga de la Virgen in Cartagena and the CGSM. Participants mentioned two social contextual drivers that related to this slow-onset degradation of environmental water quality: (i) the misuse of the mangrove and lagoon for disposal of waste from an increasing peri-urban population, particularly in La Boquilla where waste accumulation is a key cause in the reduction in mangrove extent in the Ciénaga de La Virgen (Villarte Daza et al. 2020); and (ii) the agricultural pesticide runoff resulting from land-use changes to intensive large scale commercial plantations in lagoon catchments (particularly in CGSM): ‘… the runoff brings all the poison into the lagoon’. Communities described mass fish kill events that they attributed to water pollution, changes in fluxes of seawater and freshwater and lagoon warming associated with El Niño events (as evidenced also in Whitfield et al. 2019). All communities were aware of El Niño/La Niña phenomena and the associated extreme variability in precipitation and temperature. The intense drought periods during El Niño, and the flooding during two prolonged La Niña periods in 1999 and 2010, were perceived as disastrous, and were fresh in people’s memories. Participants also expressed concerns about the current unpredictability of weather and seasons: ‘in the past, it used to rain during specific times. Nowadays, it rains whenever it wants. Things have changed’.

Exposure (Fig. 1ii) can be framed in terms of impacts on ESs. The most frequently mentioned ES, especially for CGSM, related to provisioning services. These are food and water to drink (subsistence needs), housing and lagoon mobility, with fishing as their main source of food and basic income. Fishing-related activities include buying and selling conch, oyster, clam, shrimp, crab and fish in general. Members of the community also described the use of mangroves for firewood and other uses, such as brooms and brushes, fishing gear, and, in former times, charcoal. Synergies were mentioned regarding hunting, as a means of both obtaining food and as a recreational activity (i.e. cultural ESs), but participants emphasized that it is less important now than it used to be in the past, due to declines in target species abundance and changes in legislation. Other provisioning uses from the mangrove include materials for crafts and painting, dyes and honey. The stilt house community of Nueva Venecia (20 km within the CGSM lagoon) also depends on mangroves and their surroundings for materials to build their houses, canoes and fishing gear, and residents emphasized the importance of salt for preserving fish catches. Water for irrigation was mentioned in the communities of Caño Clarín (on the northern margin of the CGSM lagoon), where livelihoods are more dependent on horticulture and subsistence farming. However, participants also stated the importance of having good mangrove cover to provide habitat for fish, birds and reptiles, and its importance in helping to regulate temperature, decrease coastal erosion, provide protection from the wind and stabilize coastline position. The importance of the wider, off-site ecological effects of these regulating ESs was described by one participant as: ‘the mangroves are the lungs of Colombia… they also purify the water, they purify the system’.

Communities revealed, in both CGSM and La Boquilla, an important emotional-cultural attachment to their coastal environment. As most members of the communities depend on the lagoon and the surrounding ecosystem for their livelihoods, there was also a sense of ownership. They often referred to the lagoon system as their ‘company’ since ‘it generates everything we need’. These sentiments reflect a sense of belonging (i.e. the cultural ES of identity (Fish et al. 2016)): ‘I like the way I live here […] Here I have everything’; and a sense of independence: ‘unemployment is high in Colombia. But we have the lagoon that provides food, we do not depend on a company or a boss. The lagoon provides job stability and even when things are difficult, it is always there’. The CGSM was also recognized for providing a space for experiencing tranquillity and a way of living (cultural ES: experiences, Fish et al. 2016): ‘it is a quiet region, where there is no guerrilla, where you live with tranquillity, you sleep peacefully, that is perhaps why we endure all the inclemencies as floods..’. For the community in La Boquilla, the proximity of Cartagena and the continued expansion of the city towards the Ciénaga de la Virgen has provided new work opportunities, and has been a key driver in the community transitioning from its traditional fishing background (‘we were born and raised in fishing’) to either eco-tourism operations and/or office or hotel work in the nearby urban developments. However, although the importance of the ecosystem in providing food and resources has been declining, participants in the focus group in La Boquilla still expressed a strong affinity towards the ecosystem. They perceived that their role was shifting towards one of guardianship and that the community has a vital role in preserving local knowledge about the ecosystem, in order to ensure its conservation (i.e. the cultural ES of capacity (Fish et al. 2016)): ‘some people study this ecosystem just because it is a job for them. For us it is our patrimony. We feel the ecosystem’.

### Community Perceptions of Sensitivity to Environmental and Climatic Pressures

The high natural resource dependency in these communities was found to be a driver of these coastal communities’ sensitivity (Fig. 1ii) to extreme weather events and slow-onset environmental degradation. As identified above, there is a narrow range of resources—farming and fishing—upon which the communities rely for their continued livelihood. An official from the Colombian National Natural Parks summarised, ‘if the Ciénaga Grande is doing well… the communities that are here will live supremely well. But if it is doing badly, obviously they are going to be doing badly as well’. An example of this close association between livelihoods and the lived-in ecosystem is provided by the 95% reduction in oyster (*Crassotrea rhizophorae*) populations due to changing rates and patterns of sedimentation during the period 1995–1996, following works to improve water connectivity of the lagoon with the Magdalena River. The harvest of oysters declined and the income of fishers was reduced by 18% as a consequence (Vilardy et al. 2011; Zamora-Bornachera and Meza-García 2013). Dependency on small-scale local fisheries is manifested by its great cultural significance and its critical economic importance, contributing to income, jobs and food security: ‘we are close, thanks to God, to the lagoon to be able to eat […] the lagoon gives us job stability […] it is always there’. Moreover, the participants reported that the lack of transferable skills, which could be used in other occupations, compounded by low levels of education, is a key sensitivity-vulnerability factor that severely limits their livelihood and mobility alternatives. In 2009, 57.5% of people aged 15 and above living in Nueva Venecia could neither read nor write (Aguilera-Díaz 2011): ‘now to get employed…, you need many requirements, having some education is the main one. You need all the documents and if you do not have them, there is no work’. Torres-Guevara et al. (2016) show that this can also lead to more unsustainable fishing practices. Community members described the importance of traditional local in-depth knowledge and social capital as key pathways in allowing them to continue their dependency with the natural environment and their cultural roots, as evidenced in Carrasquilla-Henao et al. (2019). Thus, they know where, and under what conditions, shellfish can be found in abundance, which fish species are more affected by droughts, how the different signs of nature should be read and how clamshells can also be used to enhance plant growth or to make mosaics. This in-depth knowledge is perceived as reducing sensitivity to environmental shocks. As one of the community members highlighted, ‘we have learnt to adapt to everything, to all the adversities that we can have in this area’. The majority of the studied communities are characterized by unity and strong social capital—‘those of us here are family’—which is manifested in the members’ willingness to support each other in times of need by sharing food, or shelter. Participants recalled memories of how they self-organised to address intense flooding events, with the strong sense of belonging to a place providing an extra ‘safety net’.

Communities felt that their dependency on the ecosystem was not understood by the authorities, a key driver that magnifies their sensitivity. They perceived a general lack of environmental awareness on behalf of the authorities of how governmental decisions in the past had altered the system (e.g. through public infrastructure developments), and expressed an overwhelming feeling of abandonment by the state. As an example, according to the accounts given to the authors by local community members, the last Colombian President who visited the CGSM was Ernesto Samper Pizano (President 1994–1998) in 1996/1997. Communities in CGSM face shortages of basic services, such as water supply, sewerage and electricity (Correa De Andreis 2001; Erazo 2016). Limited access to freshwater emerged as a key problem in the focus groups with the stilt house community at Nueva Venecia who stated that they have to drink salty water because of the lack of freshwater—‘it takes 6–8 h to go to fetch freshwater on a canoe rowing, and 2.5 h return on a motorboat’. Some of these socio-economic challenges of CGSM communities are inextricably linked to their geographical isolation; however, participants perceived that their challenges are mostly linked to the institutional context, as they reside and make a living in a National Park: ‘some help from the father-our government, that remembers us, and does not make a dictatorship of everything that can be caught or not’. In this respect, some participants felt that the Park’s regulations restricted their options to enhance and diversify their livelihoods, by placing legal restrictions on certain activities such as (i) charcoal extraction; (ii) the use of fishing gear, such as *zangarreo* and *boliche*, which have been shown to increase catch rates of smaller, shorter-lived and lower trophic level species (Ruedo and Defeo 2003) and (iii) diversion of the rivers that flow into the wetlands, or clogging of canals to facilitate crop irrigation. It was clear that these opposing pressures can lead to tensions between official authorities responsible for management and conservation and community members trying to access and use natural resources to provide for their families (e.g. differences in opinion between scientists and fishers on the impact of fishing activity). These tensions have also been found in terrestrial Protected National Parks in Colombia (De Pourcq et al. 2019, 2017).

### Community Perceptions of Direct Impacts from Environmental and Climatic Pressures

Communities perceived that existing slow-onset environmental pressures, such as increasing salinity of the estuary, the erosion and salinization of soils and pollution were driving trajectories in their impacted experiences (Fig. 1iii) towards higher competition for natural resources (fish); causing declining incomes (from fishing, husbandry or farming), impacting biodiversity and limiting access to basic resources such as freshwater for domestic use. Health impacts due to skin diseases and changing diets were also mentioned, especially by those involved with fishing in mangrove areas. Exposure to multiple stressors has created a general pessimistic perception about the future of the lagoon, based on a gradual deterioration in its condition. This view was shared among all participants and emphasizes the centrality of the ecosystem in the daily lives of the community: ‘if the mangroves and fish are deteriorating on a daily basis because the habitat is reducing, it is like if your house is crumbling down…each day we live worse. This is affecting us also economically because our production is being reduced’. Indeed, this is a view found consistently across multiple fishing villages within the CGSM (Carrasquilla-Henao et al. 2019). The loss of biodiversity has immediate direct effects on livelihoods but also contributes to the perceived decline in cultural values. Older focus group participants were saddened that they would no longer be able to pass down their in-depth knowledge and cultural ecosystem identity to future generations due to the loss of biodiversity: ‘our grandchildren no longer know or recognize certain fish species because they are disappearing. Flora and fauna are declining. Even today it is very rare to see some of the large reptiles, like the caiman aguaya’. Moreover, environmental degradation is changing the demographic structure of communities. The decline in natural resources means that some community members, especially the younger ones, are migrating to nearby towns and cities because they are no longer able to sustain a local livelihood. The declining proportion of younger people in the communities, and hence the reduction in opportunities for inter-generational transfer of knowledge and tradition, reinforces the impact of biodiversity loss in eroding cultural knowledge and traditions, which, in turn, impacts on the cultural value of these mangrove-fringed lagoons.

In contrast, impacts associated with climatic variability associated with El Niño and La Niña (also included in Fig. 1iii) were perceived to have immediate and short-term effects relating mainly to livelihoods (living and working conditions). Those participants who cited erratic rainfall as a climatic stressor, indicated that it affects their horticultural production, fishing activities and tourism operations and leads to lower yields and income; it also causes flooding of houses and roads, loss of electricity, and makes it difficult to dry wood for fuelwood. Excessively rainy periods impact also on the community’s physical health, for example, causing increases in infections associated with the higher presence of mosquitoes associated with intense flooding (Moreno 2006). These conditions also affect people’s mental health, leading to demoralizing effects associated with the difficulty of going out on the lagoon in dangerous storm conditions: ‘the problem is that we cannot work in those conditions. We cannot go out and fish or bring food. We cannot do anything’; ‘the water level rises in the swamp, together with the stagnant water, which if gets into the swamp kills the fish stocks’. There is also the trauma of seeing ‘everything (houses, farming fields,…) being destroyed, which takes months to rebuild’. However, participants also recognized the importance of freshwater influxes into the mangrove system and stated that after the heavy rain, the abundance of fish increased: ‘whenever it rains, you can see the change…how the ecosystem is getting better…. Fish stocks increase and trees grow’. In fact, periods of intense drought were perceived as having a more detrimental impact on livelihoods than periods of heavy rainfall. During droughts, participants described a pattern of substantial mangrove die-back, small fish catches, high fish natural mortality and lack of other provisioning services, including crops, honey and hunting. The increase in the salinity of water results in a lack of available freshwater for human consumption, irrigation and animal consumption. Higher temperatures and less rain also lower water levels in the lagoon, and transportation across the lagoon becomes more difficult. Finally, participants recalled an emerging risk derived from an increase in the number of impacts associated with the wildfires during the 2015/2016 El Niño.

### Community Perceptions of Responses to Environmental and Climatic Pressures

The communities in La Boquilla and CGSM showed similar responses (Fig. 1iv) in relation to the perceived pressures from environmental stressors and climate variability. They appear resigned to variability in environmental conditions: ‘we settle with whatever nature does’; ‘what are we going to do? We are satisfied with whatever God provides us’. Active responses to cope with environmental change include maintaining and opening channels to sustain irrigation, changing fishing locations, techniques and production processes (e.g. greater reliance on dried rather than fresh fish), relying on family financial savings and support from other community members during downturn periods, or temporarily migrating to pursue alternative livelihoods. For some community members, what had been cyclical moves to Cartagena and Barranquilla in ENSO periods of drought-like or flooding-like conditions have become permanent moves.

More specifically, coping mechanisms for ENSO episodes can be placed in three categories. First are the responses that moderate potential damage of the hazard by modifying existing livelihood practices through changes in behaviour or equipment. For instance, (a) during extreme floods in La Niña episodes, responses included moving to a dry neighbouring house, sleeping in their canoes or temporarily relocating to a nearby city (e.g. Barranquilla, Soledad, Malambo, Cartagena); or (b) for those living in the stilt house villages at the CGSM, building a second (or even third) floor (tambo) onto their homes, which they then dismantle once the water level decreases or, in the same way, (c) those making a living from harvesting clams cope with rise in water level by lengthening the stilts (zanco) that they use to move around in the water. Second, there are responses that devise alternative livelihood practices based on existing knowledge and experiences. Hence, responses are dominated by an individual’s ability to be flexible and diversify their source of livelihood: ‘one cannot let himself starve, we find another activity. We had to abandon all the work in agriculture and survive making crafts’. Third, communities’ responses also included the acquisition of new knowledge: ‘those of us who did not know how to fish, we learned, and those who did not know about crafts also learned, because it was a way to survive in the time of that flood’. For example, fishers learn to harvest alternative fish species, use different fishing gear and change the areas where they fish according to environmental and climatic conditions.

Communities viewed extreme weather events as an impetus to learn from past experiences, re-evaluating current practices and developing strategies that contribute to their preparation for future extreme events, which ranged from, as mentioned above, altering their houses, to altering irrigation channels and manually raising the level of agricultural fields. This knowledge on adaptive learning practices were reported to spread through the community: ‘after returning to my house and fields from a period of heavy flooding, I started to raise the field, taking soil from one area and bringing it nearby, to raise the level. The flood level from that particular difficult year showed me how high it could get… I started raising the level for the future and others also followed’. Similarly, in Puerto Caimán, oyster (*Crassostrea rhizophorae*) was previously harvested as a primary resource, but the species declined sharply in the late 1990s following a combination of El Niño in 1995–1996, La Niña between 1998 and 2000, changes in salinity and an increase in sedimentation (Rueda and Defeo 2003; Rueda et al. 2011). In response to this oyster decline, the fishers modified their fishing techniques to catch shrimp and crab, and relocated to other sites to maximize catch returns. Some participants indicated that they had been, or became part of, previous or existing development agency initiatives to build their skills and abilities, in order to develop parallel economic activities that enabled income diversification, such as eco-tourism, honey collecting or fish breeding in nurseries. Participation in such initiatives can be considered also as a long-term adaptation strategy. Nevertheless, the majority of donor-funded projects and initiatives were viewed by participants to be unsustainable, with a lack of monitoring and follow-up, and a failure to reflect real needs and local socio-economic dynamics.

## Discussion: Needs and Options to Strengthen Adaptive Capacity

This study was designed to develop a deep understanding of the vulnerability (exposure, sensitivity, impacts and responses; Fig. 1) of tropical coastal communities in Colombia to extreme weather events and slow-onset environmental degradation, based on communities’ long-term memories and experiences. Our results show that the La Boquilla and CGSM communities have an in-depth knowledge of the status of environmental assets of their mangrove-fringed lagoons and the dynamics of lagoonal change, related to both natural and social pressures. Communities recognized the need for investing in human capital, for example though education, to reduce their exposure and sensitivity to environmental and climatic pressures. They reported a lack of institutional effort by governmental agencies to support their way of living and restore the ecosystem on which their livelihoods depend, causing a perceived feeling of abandonment by the state. Participants identified a wide range of impacts, some immediate and short term (e.g. flooded crops and houses), others as long-term effects of social-ecological interactions (e.g. declining income capacity, loss of inter-generation knowledge exchange), of which some emerge as cascading effects (e.g. loss of biodiversity driving the decline in culture and knowledge). The communities’ flexibility to switch or rely on multiple responses to accommodate the severe fluctuations between droughts and floods over ENSO episodes was demonstrated through: (i) switching between occupations (e.g. fishing v. eco-tourism); (ii) changing fishing gears, techniques or locations to adapt to changes in species abundance or habitat destruction; (iii) re-building housing and agricultural fields at higher levels after flooding; (iv) developing skills providing alternative temporary income or subsistence livelihoods (e.g. craft making) and (v) short-term mobility, with people migrating to nearby cities (Barranquilla or Santa Marta) in order to diversify their income and exploit opportunities outside the area. Finally, our findings show that these communities generally manage climate variability in a reactive, short term, and mostly unplanned manner, with some activities reinforcing system deterioration. Nevertheless, participants were able to recognize their successes and failures, forming an experiential and reflective knowledge system that can generate feedback loops of learning, reinforcing and enhancing adaptive capacity (Fig. 1).

These findings have further wider significance when evaluating their implications regarding the needs and options for this long-term adaptation.

First, the experiences by the coastal communities investigated here are useful in illustrating the challenges of selecting the responses that can best enhance adaptive capacity and the avoidance of behaviours that can lead maladaptive outcomes (Smit and Wandel 2006; Narváez et al. 2008), which may lock ecological–economic systems into an undesirable state (Cinner 2011; Fidelman et al. 2013). For example, in the context studied here, changing to certain fishing practices has the potential to over-exploit particular species and cause deterioration of essential fish habitat. These actions can lead to lower catches per unit effort that, in turn, lead to increase of fishing effort in order to make up for lower income, exacerbating the pressure on fish stocks, compromising future livestock opportunities, and increasing the sensitivity to natural hazards. There is some indication that this downward spiral may already be occurring in the CGSM since, despite perceived declines in ecosystem health and fish stocks, the overall capture rate from the lagoon has remained relatively reliable since 2008 (Whitfield et al. 2019). The decline in fish catches has been compensated for by an increase in crustacean harvesting since 2000, and to a lesser extent, an increase in mollusc catches since 2002 (INVEMAR 2018; Whitfield et al. 2019). The shift to crabs (*Callinectes* sp.) has also been encouraged by the establishment, since 2000, of three crab processing plants in Puebloviejo on the north-east edge of the CGSM (Whitfield et al. 2019). Thus, socio-economic factors are interacting with environmental ones to change the state of fisheries in the CGSM. Changing agricultural ‘coping’ practices can also exacerbate environmental problems. For example, the practice of raising field levels and moving soil may have an effect on the stability of the river bed, affect water pathways and residence times in the lagoon and increase rates of sedimentation. In turn, these may affect the ecological health of the lagoon, as has been shown in other locations (Cramer and Hobbs 2002), thereby exacerbating the communities’ exposure to environmental degradation.

Second, coping responses may not be adaptive in the long term. Our findings suggest, consistent with the literature, that households that have the ability for short-term mobility are more resilient to livelihood shocks associated with extreme weather events than those that lack this ability (Tebboth et al. 2019). However, when people migrate permanently, this displacement can lead to broader demographic, social and cultural impacts in coastal communities, exacerbating their vulnerability (Curran 2002). Moreover, not everyone is able to move, and the lack of assets, principally financial but also, importantly, a lack of formal education, is a key factor that prevents the use of mobility as a coping strategy for many individuals; thus, those that remain are likely to be the poorest, uneducated and most vulnerable members of society. This dynamic highlights the importance of combining scientific knowledge with institutional support and social inclusivity in conservation management and adaptation planning (Berman et al. 2012; Maru et al. 2014; Cinner et al. 2018). This helps ensure that environmental stressors and community responses can be managed together, that conflicts between people and authorities can be avoided, and that short-term coping responses open up, rather than constrain, future opportunities for adaptation.

Third, the sense of abandonment, as reported for the studied communities, can lead to mistrust. This can create consequent significant institutional barriers, a central cause of ineffective adaptation (Næss et al. 2005; Lebel et al. 2011). Increased communication between communities and formal institutions are essential to build trust (e.g. Raymond and Robinson 2013; Saavedra-Diaz et al. 2015; Matera 2016), and the coproduction of knowledge, in a site- and context-specific way, are required to build effective adaptive capacities for risk reduction to extreme weather events (Glaas et al. 2010; Reyers et al. 2015; Thomas et al. 2018). In CGSM, the social capital was severely damaged due to the atrocities of La Violencia, and even when collectives are formed to represent local interests, these often get disillusioned in their constant and unsuccessful battles with the authorities; they are excluded from the decision process in designing and delivering initiatives, which affect their communities (García Acuña 2012). In a natural resource management context, this feeling of exclusion from decision-making is likely to undermine the success of policy initiatives implemented through National Park regulations. These are aimed at protecting the sustainable use of natural resources but may require modifications to behavioural norms or traditional practices now deemed to be illegal. Experiences globally show that a proactive approach combining institutional and policy solutions with operational partnership agreements, integrating the shared knowledge of multiple interest groups and a continuous re-evaluation of current practices and the co-development of long-term needs, options and responsibilities, is the best approach for managing long-term adaptation to environmental shocks and stressors (Shiferaw et al. 2014; Panditharatne 2016). We contend that engaging and empowering communities in these areas to work more closely with the range of formal public institutions that share the burden in multiple natural hazard risk management, and creating mutual trust, rather than antipathy, is critical. In the future capacities of these institutions should be empowered to improve the state of these coastal lagoon ecosystems and, to ensure that current practices do not exacerbate existing problems and close off future adaptation options, to reduce the vulnerability of the communities themselves.

## Conclusions

Our analysis has emphasised not only the importance of interacting natural and social drivers in affecting vulnerability but also the interconnections between different types of environmental risks. One specific example highlighted by the communities under study is the interaction between slow-onset sea-level rise and reduced freshwater into the lagoon due to changes in infrastructure and agriculture in the catchment. The combined effect is to exacerbate problems of salinity, eutrophication and oxygen in the lagoon, with knock-on implications for fishery stocks, livelihoods and consequent direct and indirect impacts on physical and mental health. The ESs framing of the communities’ experiences facilitated the conceptualisation of these types of complex management challenges as trade-offs between the livelihoods of different sectors of the community (e.g. lagoon fishery, coastal fishery, farmers or eco-tourism). It also highlighted the underpinning role of the ecosystem in the interactions between environmental and climatic risks and coping responses (Elwell et al. 2018), and an understanding that multiple environmental and social risks need to be managed coherently to reduce vulnerability (Simpson et al. 2021). However, the complex pathways, feedbacks and trade-offs present significant challenges for local communities in terms of how to respond to reduce both short- and long-term vulnerabilities.

Our study provides an example of how proactive attitudes, in-depth ecosystem knowledge and adaptation learning processes assist coastal poor communities to cope with difficult times. However, the communities’ responses are mostly shaped by short-term gains, and weak institutional support in mitigating, preparing and helping these communities to respond to, and recover from, natural and climatic hazards. As the planet warms, and sea-level rises, understanding how the interactions of physical, climatic, environmental and institutional mechanisms re-organize behavioural responses to these threats needs to complement the information available from quantitative and qualitative models of social vulnerability, to offer predictions of potential thresholds that can ‘lock-in’ communities in undesirable socio-ecological states. Future development of governance for climate change adaptation needs to tackle all these dimensions of vulnerability.
